# Response‐adaptive designs for binary responses: How to offer patient benefit while being robust to time trends?

**DOI:** 10.1002/pst.1845

**Published:** 2017-12-19

**Authors:** Sofía S. Villar, Jack Bowden, James Wason

**Affiliations:** ^1^ MRC Biostatistics Unit Cambridge Institute of Public Health Cambridge UK; ^2^ MRC Integrative Epidemiology Unit University of Bristol Bristol UK

**Keywords:** clinical trials, power, response‐adaptive randomisation, randomisation test, type I error

## Abstract

Response‐adaptive randomisation (RAR) can considerably improve the chances of a successful treatment outcome for patients in a clinical trial by skewing the allocation probability towards better performing treatments as data accumulates. There is considerable interest in using RAR designs in drug development for rare diseases, where traditional designs are not either feasible or ethically questionable. In this paper, we discuss and address a major criticism levelled at RAR: namely, type I error inflation due to an unknown time trend over the course of the trial. The most common cause of this phenomenon is changes in the characteristics of recruited patients—referred to as patient drift. This is a realistic concern for clinical trials in rare diseases due to their lengthly accrual rate. We compute the type I error inflation as a function of the time trend magnitude to determine in which contexts the problem is most exacerbated. We then assess the ability of different correction methods to preserve type I error in these contexts and their performance in terms of other operating characteristics, including patient benefit and power. We make recommendations as to which correction methods are most suitable in the rare disease context for several RAR rules, differentiating between the 2‐armed and the multi‐armed case. We further propose a RAR design for multi‐armed clinical trials, which is computationally efficient and robust to several time trends considered.

## 1 INTRODUCTION

Randomised controlled trials (RCTs) are considered the gold standard approach to learn about the relative efficacy of competing treatment options for evidence‐based patient care. The information provided by an RCT can subsequently be used to better treat future populations. Traditionally, patients are allocated with a fixed and equal probability to either an experimental treatment or standard therapy arm. We will refer to RCTs implemented in this way as incorporating complete randomisation (CR). However, there is generally a conflict between the individual benefit of patients in the trial and the collective benefit of future patients. Complete randomisation, by definition, does not provide the flexibility to alter the allocation ratios to each arm, even if information emerges that breaks the initial trial equipoise. This conflict becomes more acute in the context of rare life‐threatening conditions, because the trial participants generally make up a sizeable proportion of the total patient population.

Response‐adaptive randomisation (RAR) offers a way of simultaneously learning about treatment efficacy while also benefiting patients inside the trial. It achieves this by skewing allocation to a better performing treatment, if it exists, as data are accrued. When RAR rules are used in a multi‐armed trial, they also increase the probability of finding a successful treatment and speed up the process of doing so.(1, 2)

However, RAR is still infrequently used in practice. One of the most prominent recent arguments against its use is the concern that the false positive error rate (or type I error rate) may not be controlled at the nominal level.^3^ This can easily occur if the distribution of patient outcomes changes over time and the traditional methods of analysis are used.^4^ One such example is when the underlying prognosis of patients recruited in the early stages of a trial differs from those recruited in the latter stages. This is often referred to as “patient drift.” Karrison et al^5^ investigate the type I error inflation induced by various RAR rules implemented within a 2‐armed group sequential design with a binary outcome in which, depending on the observed value of the corresponding *z*‐statistics, the next group of patients is allocated in one of 4 possible fixed ratios *R*(*z*). They show that if all success rates increase by 0.12 over the course of a study with 3 interim analysis, the type I error rate achieved by a group sequential design is “unacceptably high,” with the inflation being worst for the most aggressive RAR rules.

Time trends are more likely to occur in studies that have a long duration. Consider, for example, the Lung Cancer Elimination (BATTLE)‐1 phase II trial which recruited patients for 3 years (2006‐2009). It was found that more smokers and patients who had previously received the control treatment enrolled in the latter part of the study compared to the beginning of the study.^6^ Trials that last more than 3 years will often be required for rare diseases because of the recruitment challenge. It is also exactly for this case where the use of RAR can be most desirable as the trial patients represent a higher proportion of the total patient population and the suboptimality gap of traditional RCTs—in terms of overall expected patient benefit—increases as the prevalence of the disease decreases.^7^

There has been little work in the literature considering the impact of time trends on different RAR rules. In Coad,^8^ sequential tests for some RAR rules that allow for time trends are constructed while estimation and the issue of bias within this context are addressed in another study.^9^ In Rosenberger et al,^10^ a covariate‐adjusted response‐adaptive mechanism for a 2‐armed trial that can take a specific time trend as a covariate is introduced. In the trial context investigated by Karrison et al,^5^ an analysis stratified by trial stage eliminates the type I error inflation induced by a simple upward trend of all the success rates. A similar stratified analysis is used in Coad.^11^ More recently, Simon and Simon^4^ considered broad RAR rules for the 2‐armed case and proposed a randomisation test to correct for type I error inflation caused by unknown time trends of any type. There have been several recent papers comparing different classes of RAR rules under various perspectives (see, eg, previous studies(12, 13, 14, 15)), yet most of these do not examine the effects of time trends as done in Coad.^11^ An exception to this is Thall et al^3^ in which type I error inflation under time trends is pointed out as an important criticism of RAR. However, their paper only investigates a special class of RAR (based on regular updates of posterior probabilities). In this paper, we identify and address a number of unanswered questions that we describe below, including the study of the multi‐armed case.

If one is considering designing a clinical trial using a particular RAR rule, then a fundamental question to consider is how large the temporal change in the trial data has to be to materially affect the results. In Section 2, we address this question for a representative selection of RAR procedures.

If the possibility of a large drift occurring during the trial is a concern and a RAR scheme is being considered for designing such a trial, then subsequent and related questions are as follows: Do any “robust” hypothesis testing procedures exist that naturally preserve type I error in the presence of an unknown time trend? Should these procedures be different for 2‐armed and for multi‐armed trials? Should they differ depending on the RAR rule in use? And, finally, what is their effect on statistical power? Sections 2 and 3 address these questions for different RAR procedures. In Section 4, we consider whether time trends can be effectively detected and adjusted for in the analysis and how extended modelling approaches for modelling a time trend compare to model‐free approaches to control for type I error. In Section 5, some conclusions and recommendations for addressing this specific concern are given.

## 2 RAR RULES, TIME TRENDS, AND TYPE I ERROR RATES

In this section, we assess the impact of different time trend assumptions on the type I error rate of distinct RAR procedures. We assume that patients are enrolled in the trial sequentially, in groups of equal size *b* over *J* stages. We do not consider monitoring the trial for early stopping, and therefore, the trial size is fixed and equal to *T*=*b*×*J*. We have omitted it the possibility of early stopping in this paper to isolate the effects of an unaccounted for time trend in a trial design using RAR. Patients are initially allocated with an equal probability to each treatment arm. After the first interim analysis, allocation probabilities are updated based on data and according to different RAR rules. In a real trial, this initial CR start‐up phase could be replaced by a restricted randomisation phase (eg, a permuted block design) to minimise sample imbalances and improve the subsequent probabilities updates.^16^ For simplicity of presentation, we consider a binary outcome variable *Y*_*i*,*j*,*k*_ for patient *i* allocated to treatment *k* at stage *j* (with *Y*_*i*,*j*,*k*_=1 representing a success and *Y*_*i*,*j*,*k*_=0 a failure) that is observed relatively quickly after the allocation. An example might be whether a surgery is considered to have been successful or not.

We consider a trial with *K*⩾1 experimental arms and a control arm and assume that every patient in the trial can only receive one treatment. We will also assume that for every *j*<*J* before making the treatment decisions for the (*j*+1)th block of patients, the outcome information of the *j*th block of patients is fully available. Patients in block *j* are randomised to treatment *k* with probability *π*_*j*,*k*_(for *j*=1,…,*J* and *k*=0,1,…,*K*). For example, a traditional CR design will have *π*_*j*,*k*_=1/(*K*+1)∀*j*,*k*. Patient treatment allocations are recorded by binary variables *a*_*i*,*j*,*k*_ that take the value 1 when patient *i* in block *j* is allocated to treatment *k* and 0 otherwise. Because we assume that patients can only receive one treatment, we impose that $\sum_{k=0}^{K}a_{i,j,k}=1$ for all *i*,*j*. We denote the control treatment by *k*=0. Updating the allocation probabilities after blocks of patients rather than after every patient makes the application of RAR rules more practical in real trials.^17^

An appropriate test statistic is used to test the hypotheses that the outcome probability in each experimental treatment is equal to that of the control. That is, if we let *P* *r*(*Y*_*i*,*j*,*k*_=1|*a*_*i*,*j*,*k*_=1)=*p*_*k*_, then we consider the global null to be *H*_0,*k*_:*p*_0_=*p*_*k*_ for *k*=1,…,*K*. Generally, any sensible test statistic will produce reliable inferences if the outcome probability in each arm conditional on treatment remains constant over the course of the trial. If this is not the case, then the analysis may be subject to bias. To illustrate this, we shall assume the following model for the outcome variable *Y*

(1) $$\text{Logit}\left[Pr(Y_{i,j,k}=1|Z_{i,j},a_{i,j,k}=1)\right]=\left\{\begin{array}{cc} \beta_{0}+\beta_{t}t_{j}+\beta_{z}Z_{i,j} & k=0\\ \beta_{0}+\beta_{t}t_{j}+\beta_{z}Z_{i,j}+\beta_{k} & k⩾1 \end{array},\right.$$

where *t*_*j*_=(*j*−1), *Z*_*i*,*j*_ is a patient‐level covariate (eg, a binary indicator variable representing whether a patient characteristic is present or absent), and therefore, *β*_*t*_ is a time trend effect, *β*_*z*_ is the patient covariate effect, and *β*_*k*_ is treatment's *k* main effect. We shall assume that *Z*_*i*,*j*_∼*B* *e* *r* *n*(*q*_*j*_) and define $\text{Expit}(u)=\frac{exp(u)}{1+exp(u)}$. Furthermore, we shall assume that the global null hypothesis is true, meaning *H*_0,*k*_ holds for *k*=1,…,*K*, or equivalently *β*_1_=⋯=*β*_*k*_=0. Patients with *Z*_*i*._=1 will have success rate when allocated to arm *k* equal to Expit(*β*_0_+*β*_*t*_ *t*_*j*_+*β*_*z*_) while patients with a negative value *Z*_*i*._=0 will have a success rate of Expit(*β*_0_+*β*_*t*_ *t*_*j*_).

If the covariate variable *Z* is unobservable then when analysing the data, response rates will in effect be marginalised over *Z* as follows:

(2) $$\begin{array}{ccc} Pr(Y_{i,j,k}=1|a_{i,j,k}=1) & =\sum_{f=0}^{1}Pr(Y_{i,j,k}=1|Z_{i,j}=f,a_{i,j,k}=1)Pr(Z_{i,j}=f) & \\ & =\text{Expit}(\beta_{0}+\beta_{t}t_{j})(1-q_{j})+\text{Expit}(\beta_{0}+\beta_{t}t_{j}+\beta_{z})q_{j}. \end{array}$$

Assuming that equal numbers of patients are recruited at each of *J* stages, then the mean response rate in arm *k* will be

(3) $$Pr(Y_{i,.,k}=1|a_{i,.,k}=1)=\frac{1}{J}\sum_{j=1}^{J}Pr(Y_{i,j,k}=1|a_{i,j,k}=1).$$

The inclusion of *t*_*j*_ and *Z*_*i*,*j*_ allows us not only to introduce time trends of different magnitude but also to describe 2 distinct scenarios that are likely to be a concern in modern clinical trials: *changes in the standard of care* (scenario 1)—or changes in the effectiveness of the control treatment—and *patient drift* (scenario 2)—or changes in the baseline characteristics of patients. Under model (1), we shall consider that a case of scenario 1 occurs if *β*_*t*_≠0 while *β*_*z*_=0 whereas an instance of scenario 2 happens if *β*_*z*_≠0 while *β*_*t*_=0 and *q*_*j*_ evolves over *j*.

In this section, we consider the global null hypothesis by setting *β*_*k*_=0 for all *k* for both scenarios. In Sections 3.3 and 4, we consider extensions of these scenarios where *β*_*k*_>0 for some *k*⩾1. Specifically, we consider alternative hypotheses of the form *H*_1,*k*_:*p*_*k*_−*p*_0_=Δ*p*>0 for some *k*⩾1 with the treatment effect Δ*p* defined as Δ*p*=*P* *r*(*Y*_*i*,.,*k*_=1|*a*_*i*,.,*k*_=1)−*P* *r*(*Y*_*i*,.,*k*_=1|*a*_*i*,.,0_=1).

### 2.1 RAR procedures considered

Many variants of RAR have been proposed in the literature. However, different RAR procedures often perform similarly, because they obey the same fundamental principle. *Myopic* procedures determine the “best” allocation probabilities for the next patient (or block of patients) according to some criteria based on the accumulated data (on both responses and allocations) up to the last treated patient. *Non‐myopic* procedures consider not only current data but also all possible future allocations and responses to determine the allocation probability of every patient (or block of patients) in the trial (see chapter 1 in Hu and Rosenberger^18^). Furthermore, RAR procedures can be considered to be *patient benefit oriented* if they are defined with the goal of maximising the exposure to a best arm (when it exists). Additionally, RAR procedures can also be defined with the goal of attaining a certain level of statistical power to detect a relevant treatment effect, thus being *power oriented*. Response‐adaptive randomisation rules that score highly in terms of patient benefit generally have lower power.

Thus, to illustrate these 4 types of rules, we focus on the following RAR rules: “Thompson sampling” (TS) *(myopic patient benefit oriented)*, “Minimise failures given power” (RSIHR) *(myopic power oriented)*, the “forward‐looking Gittins index rule” (FLGI) *(non–myopic patient benefit oriented)*, and its controlled version, the ‘controlled forward‐looking Gittins index rule” (CFLGI) *(non–myopic power oriented)*. A short summary of these approaches is now given; for a more detailed description, see Villar et al.^19^

(a) TS: Thompson^20^ was the first to recommend allocating patients to treatment arms based on their posterior probability of having the largest response rate.
  We shall compute the TS allocation probabilities using a simple Monte Carlo approximation. Moreover, we shall introduce a tuning parameter *c* defined as $\frac{(\;j-1)\times b}{2T}$ where (*j*−1)×*b* and *T* are the current and maximum sample size, respectively. This parameter tunes the *aggressiveness* of TS allocation rule based in the accumulated data so that the allocation probabilities become more skewed towards the current best arm only as more and more data accumulate. Notice that TS is essentially the only class of RAR considered in Thall et al.^3^
(b) RSIHR: Rosenberger et al^21^ proposed and studied an optimal allocation ratio for 2‐armed trials in which the allocation probability to the experimental arm is defined as
  (4) $$\pi_{j,1}=\frac{\sqrt{p_{1}}}{\sqrt{p_{0}}+\sqrt{p_{1}}}.$$
  This allocation procedure is optimal in the sense that it minimises the expected number of failures for a fixed variance of the estimator under the alternative hypothesis that there is a positive treatment effect Δ*p*=*p*_1_−*p*_0_>0. The optimal allocation ratios that extend Equation 4 for the general case in which *K*>1 do not admit a closed form; however, numerical solutions can be implemented as in Tymofyeyev et al.^22^
  In practice, the allocation probabilities may be computed by plugging in a suitable estimate for the *p*_*k*_’s using the data up to stage *j*−1. In our simulations, we implemented the optimal allocation ratio for RSIHR using the doubly‐adaptive biased coin design. Specifically, we used Hu and Zhang's randomisation procedure with allocation probability function given by equation (2) in Tymofyeyev et al^22^ and *γ*=2. Notice that we estimated the success rate parameters from the mean of its prior distribution for the first block of patients (when no data were available) and the posterior mean thereafter.
(c) FLGI: In Villar et al,^19^ we introduced a block randomised implementation of the optimal deterministic solution to the *classic* multi‐armed bandit problem, first derived in other studies.(23, 24) The FLGI probabilities are designed to mimic what a rule based on the Gittins index (GI) would do. See Section 3 and Figure 1 in Villar et al^19^ for a more detailed explanation of how these probabilities are defined and approximately computed via Monte Carlo. The near optimality attained by this rule differs from the one targeted in procedure (b) in the sense that average patient outcome is nearly maximised with no constraint on the power levels that should be attained. Notice that before the introduction of this procedure based on the GI, an important limitation to the practical implementation of non‐myopic RAR rules such as those in Cheng and Berry^7^ and Williamson et al^25^ was computational, particularly in a multi‐armed scenario.
(d) CFLGI: In addition to the rule described in (c), for the multi‐armed case (ie, *K*>1), we consider a group allocation rule that, similarly to the procedure proposed in Trippa et al,^26^ protects the allocation to the control treatment so it never goes below 1/(*K*+1) (ie, its fixed equal allocation probability) during the trial.

**Figure 1:**
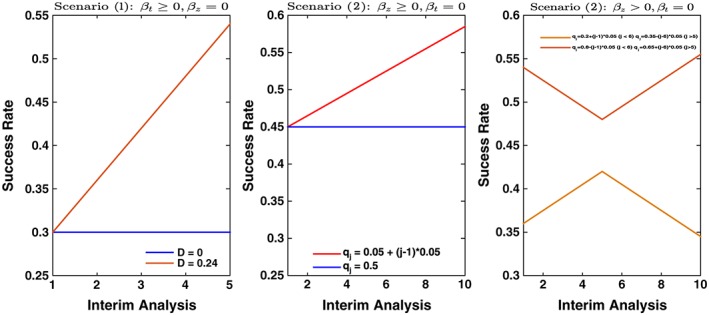
The per block success rate under different time trend assumptions plotted over time. Left plot corresponds to scenario 1 (changes in standard of care), and middle and right plots correspond to different cases of scenario 2 (patient drift)

### 2.2 Simulation results

In this section, we present the results of various simulation studies that show, for instances of scenarios 1 and 2 (described in detail below), the degree to which the type I error rate can be inflated for different RAR rules relative to a CR design. As is the usual case when comparing RAR procedures, we consider measures of efficiency (or variability) and ethical performance, assessing which one of them (if any) provides a better compromise between these 2 goals.^14^ We therefore also compute the expected number of patients assigned to the best treatment (*p* ^∗^) and expected number of patient successes (ENS). However, under the global null considered in this section, ENS and *p* ^∗^ are identical for all designs and therefore we do not report them here. In sections where we consider scenarios under various alternative hypotheses, we report patient benefit measures as well as power. Specifically, we report *p* ^∗^ and the increment in the expected patient benefit that the RAR rule considered attains over a CR design, ie, ΔENS=ENS_*RAR*_−ENS_*CR*_.

For each scenario, a total of 5000 trials were simulated under the global null and the same global null was tested. We used *z*‐statistics for testing with RAR rules (a), (b), and (d) (when asymptotic normality can be assumed) and, given that bandit‐based procedures can result in very small sample sizes for some arms, an adjusted Fisher exact test for procedure (c). The adjustment for the bandit rules chooses the cut‐off value to achieve a 5% type I error rate (as in Villar et al^27^). For multi‐armed trials, we use the Bonferroni correction method to account for multiple testing and therefore ensure that the family‐wise error rate is less than or equal to 5%. In all simulations and for all RAR rules, we assumed uniform priors on all arms' success rates before treating the first block of patients.

#### 2.2.1 Scenario 1: changes in the standard of care

The first case we consider is that of a linear upward trend in the outcome probability of the control arm. This could be the case of a novel surgery technique that has recently become the standard of care, but it requires a prolonged initial training period for most surgeons to become proficient in these complex procedures until “failure” is eliminated or reduced to a minimum constant rate. In terms of the model described in Equation 1, this corresponds to varying *β*_*t*_ with all else fixed.

Specifically, we let *β*_*t*_ take a value such that the overall time trend within the trial

$$D=Pr(Y_{i,j,.}=1|t_{j}=J-1)-Pr(Y_{i,j,.}=1|t_{j}=0)$$

varies in *D*={0,0.01,0.02,0.04,0.08,0.16,0.24}. Figure 1 (left) shows the corresponding evolution of the per block success rate of every arm over time across the scenario in which *J*=5 and for the cases of no drift (*D*=0, dark blue) and the strongest drift considered (*D*=0.24, dark red).

**Figure 2:**
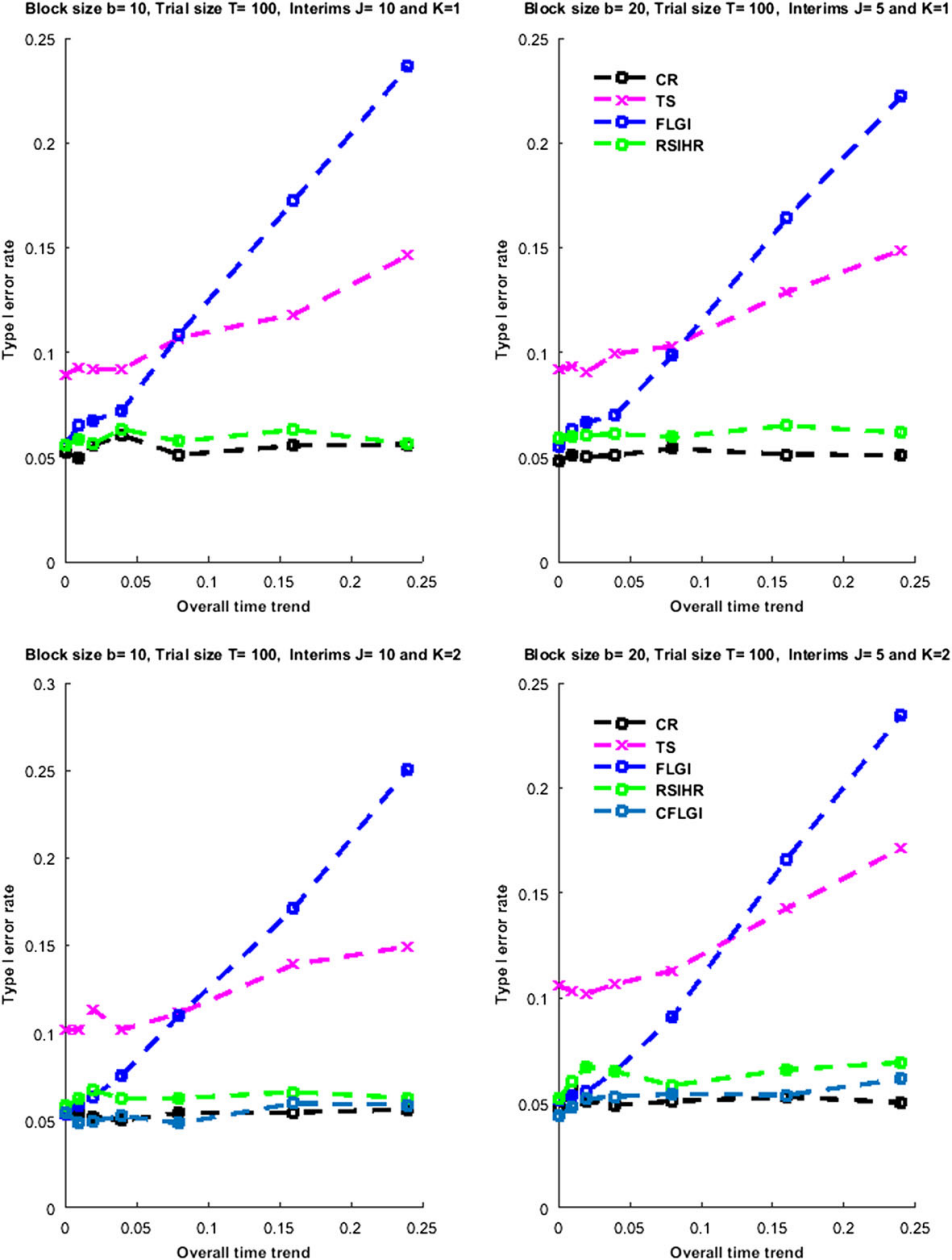
The type I error rate for scenario 1 (changes in the standard of care) under different linear time trends assumptions and different response‐adaptive randomisation rules. CR, complete randomisation; CFLGI, controlled forward‐looking Gittins index rule; FLGI, forward‐looking Gittins index rule; RSIHR, minimise failures given power; TS, Thompson sampling

Figure 2 summarises the simulation results. The top row of plots shows the results for the 2‐armed trials (ie, K=1), and the bottom row of plots shows the results for K=2. In both cases, the trial size was T=100. The value of the sample size T might be interpreted as the maximum possible sample size (ie, including a very large proportion of the patient population) in the context of a rare disease setting. The plots in the left column assume a block size of 10, and the plots in the right column assume a block size of 20. The initial success rate was assumed to be equal to 0.3 (which corresponds to β_0_≈−0.8473) for all the arms considered.

Under the assumption of no time trend (ie, *β*_*t*_=*D*=0), the test statistics used preserve the type I error rate for all designs in all cases considered, except for TS for which the false positive rate is somewhat inflated (as pointed out in Thall et al^3^). For CR, the type I error rate is preserved even when a time trend is present and regardless of the block size, number of arms, and the trend's magnitude.

The error rates for some of the RAR rules (FLGI and TS) are substantial when overall time trends are of 0.08 and more. This is because these rules are *patient benefit oriented*, ie, they skew allocation towards an arm based on data more considerably and/or earlier on in the trial. On the other hand, the RSIHR procedure, being a *power‐oriented* rule, remains practically unaffected by temporal trends in terms of type I error inflation. This very important difference in performance amongst RAR procedures has not been noted previously.

Multi‐arm allocation rules that protect allocation to the control arm, like the CFLGI, are also unaffected by type I error inflation, even for large drifts. Generally, the type I error inflation suffered by the other RAR rules seems to be slightly larger for the 3‐armed case than for the 2‐armed case.

#### 2.2.2 Scenario 2: patient drift

For this case, we imagine a simplistic instance in which patients are classified into 2 groups according to their prognosis. This occurs if, for example, *Z*_*i*,*j*_ in model (1) represents the presence or absence of a biomarker in patient *i* at stage *j*, where *Z*_*i*,*j*_=1 denotes a biomarker‐positive patient and *Z*_*i*,*j*_=0 denotes biomarker‐negative patient. Alternatively, *Z*_*i*,*j*_ can capture any other patient feature. It could, for example, represent if a patient is a smoker and previously received the control arm, which would be a relevant covariate in the BATTLE‐1 trial. Moreover, we let the recruitment rates of these 2 types of patients, ie, *q*_*j*_, vary as the trial progresses to induce the desired drift in the mix of patients over time. We will model this situation by letting *β*_*z*_>0 in (1) whilst holding all else fixed. We start by assuming that *Z* is unobserved. In Section 4.1, we explore the case where *Z* is measured and can be adjusted for.

The middle and right‐hand side plots in Figure 1 show the evolution of *P* *r*(*Y*_*i*,.,*k*_=1|*a*_*i*,.,*k*_=1) under differing patterns of *patient drift* over the course of the trial. The middle plot describes the case in which there is a linear trend in the average success rates of all arms created by the *patient drift* whereas the plot to the right considers the case of a more complex temporal evolution with the average success rates going up and then down or vice‐versa. In both cases, a trial of size *T*=200 with *J*=10 and therefore *b*=20 was considered. The success rates for all arms for the biomarker‐negative patients were *P* *r*[*Y*_*i*,*j*,._=1|*Z*_*i*,*j*_=0]=0.3 (so that *β*_0_≈−0.8473). For the biomarker‐positive group, *P* *r*[*Y*_*i*,*j*,._=1|*Z*_*i*,*j*_=1]=0.6(such that *β*_*z*_≈1.2528).

**Figure 3:**
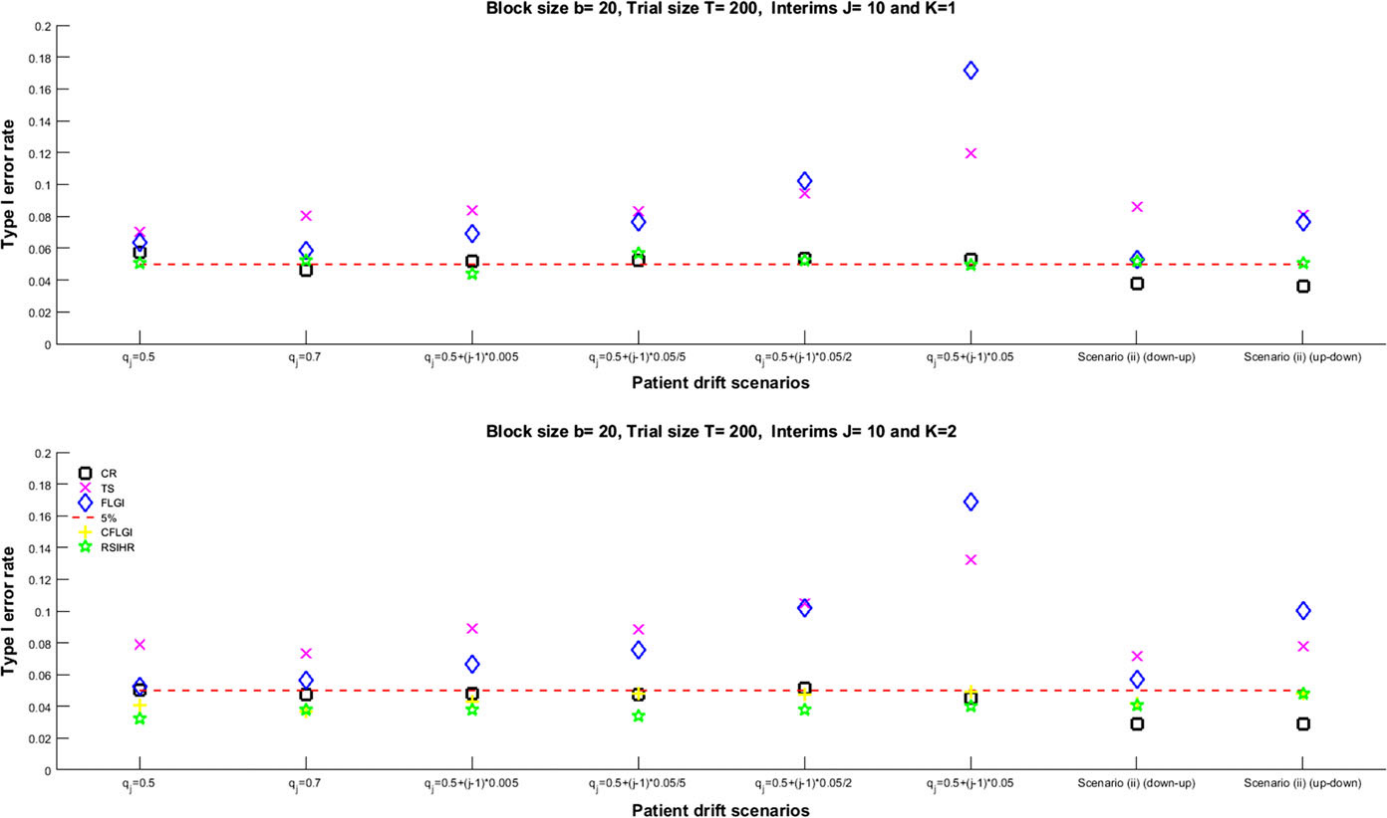
The type I error rate for different group recruitment rates assumptions under scenario 2 with β_z_≈1.2528. CR, complete randomisation; CFLGI, controlled forward‐looking Gittins index rule; FLGI, forward‐looking Gittins index rule; RSIHR, minimise failures given power; TS, Thompson sampling

Figure 3 summarises the simulation results for the cases depicted in Figure 1 (middle and right). The results show that the RAR procedures most affected by type I error inflation are those that are *patient benefit* oriented (FLGI and TS). Type I error inflation is high only for the moderately large recruitment rate evolution assumed. As before, the *power‐oriented* rules (RSIHR and CFLGI) have type I error rates that do not significantly differ from those obtained by a CR design. This further supports the argument that not all RAR procedures are equally affected by the presence of the same temporal trend.

## 3 TESTING PROCEDURES AND RAR DESIGNS ROBUST TO PATIENT DRIFT

In this section, we describe a hypothesis testing procedure for RAR rules in a 2‐armed trial context and a RAR design for multi‐armed trials that preserves type I error rates in the presence of an unknown time trend.

### 3.1 Two‐armed trials: randomisation test and the FLGI

The type I error inflation shown in scenarios 1 and 2 for some of the RAR rules is caused by the fact that the test statistics used assume every possible sequence of treatment allocations (ie, every possible trial realisation) is equally likely. For instance, this is the case for the adjusted Fisher exact test used for the FLGI in the previous sections. This assumption is not true in general, as certain allocation sequences will be highly unlikely or even impossible for some RAR procedures. This is particularly well illustrated in the case of the FLGI rule where it is possible for one of the arms to be effectively “selected” within the trial, since the probability of assigning a patient to any other arm from that point onwards is zero.

In this section, we show the results of developing and computing a test statistic, introduced in Simon and Simon,^4^ based on the distribution of the assignments induced by the FLGI under the null hypothesis. In their paper, the authors show that using a cut‐off value from the distribution of the test statistic generated by the RAR rule under the null hypothesis, and conditional on the vector of observed outcomes, ensures the control of the type I error rate (see Theorem 1 in Simon and Simon^4^). Their result applies to any RAR rule and any time trend in a 2‐armed trial; most importantly, its implementation does not require any knowledge or explicit modelling of the trend. In this paper, we have chosen to implement it for the FLGI rule, as this is the most recently proposed RAR procedure of the ones considered. Notice that type I error rate preservation under time trends by means of randomisation‐based inference for restricted randomised procedures is established in Rosenberger and Lachin.^28^ ^, section 6.10^

However, computation of the null distribution can be challenging under realistic trial scenarios, as it requires the complete enumeration of all trial histories and it is infeasible for response‐adaptive rules that are deterministic as, eg, the GI rule is. Therefore, there is a need to find ways of computing such a randomisation test efficiently for the sake of its practical implementation as well as evaluating its effect on power, which might differ across different rules.

We implement a randomisation test for the FLGI rule that is based on a Monte Carlo approximation of the exact randomisation test. More precisely, our approach does the following: for a given trial history **y**=(**y**_1_,**y**_2_,…,**y**_*J*_), where **y**_*j*_ a vector of the *b* observed outcomes at stage *j*, we simulate *M* trials under the FLGI allocation rule. The FLGI allocation ratios are updated after each block using the allocation variables *a*_*i*,*j*.*k*_ randomly generated under the FLGI rule by Monte Carlo and the observed outcome data up to that point (ie, (**y**_1_,…,**y**_*j*_)). For each of these *M* simulated trials, we compute the value of the test statistic to assemble an empirical distribution of the test statistic under the null. We can then compare the test statistic observed in the original trial to the empirical distribution, rejecting the null hypothesis at level *α* if it is more extreme than its *α* percentile for a one‐sided test (or than its *α*/2 or 1−*α*/2 percentile for a 2‐sided test). Finally, we repeat this procedure for another *N* *r* trial history replicates and report the average type I error rate achieved as well as the averages of the other ethical performance measures considered.

Table 1 shows the results from *N* *r*=5000 replicates using the approximate randomisation test for the case of scenario 1 displayed in Figure 2 (top‐right). For each trial replicate, the approximate randomisation‐based test was computed using *M*=500 simulated trials—or *resamplings*—to construct the empirical distribution function. The values of *N* *r* and *M* are the same as those used by the simulations in Simon and Simon.^4^ From Table 1, we see that the type I error rate is preserved at its 5*%* level even when the patient drift is severe. We also report *p* ^∗^ and Δ*E* *N* *S*, as defined in Section 2.

**Table 1:** The type I error rate for the approximate randomisation test from 5000 replicates of a 2‐arm trial of size T=100 using an FLGI with block size b=20 (J=5) and under the case of scenario 1 depicted in Figure 2 (top‐right plot)

*α*(s.e.)	*p* ^∗^(s.e.)	ΔENS	*D*
0.0445 (0.21)	0.501 (0.21)	0.19	0
0.0480 (0.21)	0.506 (0.22)	−0.17	0.08
0.0449 (0.20)	0.494 (0.23)	0.02	0.16
0.0445 (0.21)	0.499 (0.24)	0.23	0.24

Abbreviation: FLGI, forward‐looking Gittins index rule.

### 3.2 Multi‐armed trials: protecting allocation to control

As shown in the simulation results reported in Section 2, the RAR rules that include a protection of the allocation to the control treatment (specifically, the CFLGI) preserve the type I error rate. Matching the number of patients allocated to control to that allocated to the best performing arm has also been found to produce designs that result in power levels higher than that of a CR design (see, eg, Villar et al(19, 26)). In the next section, we report on results that indicate that this *matching* feature not only preserves the type I error rates but also ensures high power levels when using the standard analysis methods as an approximation inference method and under the presence of time trends.

Therefore, if the design of the multi‐arm trial incorporates protection of the control allocation, there appears to be no need to implement a testing procedure that is specifically designed to be robust to type I error inflation. This is another important and novel finding.

### 3.3 Protecting against time trends and its effect on power

Preserving the type I error rate is an important requirement for a clinical trial design. However, the learning goal of a trial also requires that, if a best experimental treatment exists, then the design should also have a high power to detect it. In this section, we therefore assess the power of the approximate randomisation test (for the FLGI) and the standard test (for the CFLGI).

We first explore an extension of an instance of scenario 1 in which we assume that there is a treatment effect of 0.4 (where *p*_0_=0.3 and *p*_1_=0.7; therefore, *β*_1_≈1.6946), which is maintained even in the cases where we also assume a positive time trend in the standard of care. The trial is of size *T*=150 with *J*=5 stages (so that *b*=30). Under this design, the assumed treatment effect is detected with approximately 80*%* power by the FLGI rule if there is no time trend and the adjusted Fisher test is used. If a traditional CR design is used, then the power attained is 99*%*, but the proportion of patients allocated to each arm is fixed at 1/2.

Table 2 shows the power to reject the null hypothesis as the overall time trend increases from 0 to 0.24 (ie, for *β*_*t*_∈{0,…,0.24} while *β*_*z*_=0) for a treatment effect of 0.4 (ie, for *β*_1_≈1.6946). We denote by (1−*β*_*F*_) the power level attained by the adjusted Fisher test and by (1−*β*_*RT*_) the power level when using the approximate randomisation test.

**Table 2:** Power for the approximate randomisation test from 5000 replicates of a 2‐arm trial of size T=150 using an FLGI with block size b=30 (J=5) under a case of scenario 1 with a treatment effect of 0.40

(1−*β*_*F*_)(s.e.)	(1−*β*_*RT*_)(s.e.)	*p* ^∗^(s.e.)	ΔENS	*D*
0.8086 (0.39)	0.6057 (0.48)	0.871 (0.09)	22.04	0
0.8972 (0.30)	0.6080 (0.49)	0.881 (0.04)	24.01	0.08
0.9524 (0.21)	0.6021 (0.50)	0.878 (0.05)	23.99	0.16
0.9802 (0.14)	0.5851 (0.48)	0.882 (0.03)	23.73	0.24

Abbreviation: FLGI, forward‐looking Gittins index rule.

These results show that the power of the randomisation test is considerably reduced compared to that obtained using Fisher exact test. However, the patient benefit properties of the FLGI over the CR design are preserved in all the scenarios. The improvement in patient response of the FLGI design over CR is around 30% regardless of the drift assumption.

Next, we consider the multi‐arm case by assessing the effect on power on the RAR rules that protect allocation to the control arm. To do so under different trend assumptions, we extend a case of scenario 1. We assume then that there is a treatment arm that has an additional benefit over the other 2 arms of 0.275 (where *p*_0_=*p*_2_=0.300 and *p*_1_=0.575), which is maintained even in the cases we assume a positive time trend in the success rate of the standard of care. Regardless of the trend assumption, a traditional CR design has a mean *p* ^∗^ value of 1/3 by design and detects a treatment effect of such a magnitude with approximately 80*%* power.

**Figure 4:**
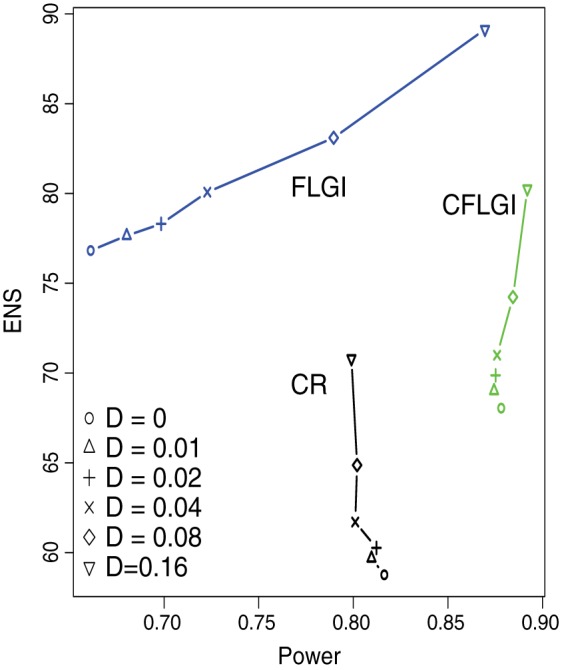
ENS‐power trade‐off of CR, CFLGI, and FLGI in 5000 replicates of a 3‐arm trial of size T=100 with block size b=20 (J=5) under a case of scenario 1 with a treatment effect of 0.275 for arm 1. CR, complete randomisation; CFLGI, controlled forward‐looking Gittins index rule; ENS, expected number of patient successes; FLGI, forward‐looking Gittins index rule

Figure 4 shows the ENS‐power levels for the designs considered. The CR design performs as predicted in terms of power and ENS. The power of the CFLGI is unaffected except for a small increase when the trend is very high. This approach attains an improvement over CR on ENS for every trend magnitude assumption considered (the improvement in ENS goes from 15.81% to 13.44% in the case of the largest assumed trend).

Figure 4 also includes the results for the FLGI for comparison. Power levels are increased when there is a positive time trend, as assumed in this case, compared to the case of no time trend. This is due to the temporal upward trend which for the FLGI rule causes an overestimation of the treatment effect. Notice that the probability in these simulations of the allocation imbalance observed in the FLGI being in the wrong direction (ie, towards inferior arms) only occurred in less than 4% of all replicates.

## 4 ADJUSTING THE MODEL FOR A TIME TREND

In this section, we illustrate the extent to which adjusting for covariates can help to reduce type I error inflation and affect power. This section also discusses the problems that can be encountered when doing this after having used a RAR procedure and how to address them.

### 4.1 Two‐armed trials

We first study covariate adjustment under instances of scenario 1. We consider a 2‐armed trial of size *T*=100 with *J*=5 and *b*=20. We shall focus on the most extreme case considered in Figure 1 in which the overall time trend was *D*=0.24 (or *β*_*t*_≈0.2719). The initial success rates of both arms were set to 0.3 (ie, *β*_0_≈−0.8473).

Parts (I) and (III) in Table 3 show the results for the estimation of the models' parameters using standard maximum likelihood estimation, when the (logistic) model is correctly specified. These results indicate, perhaps unsurprisingly, that for both designs, the treatment effect is found to be significant in less than 5% of the 5000 trials, which suggests that by including a correctly modelled time trend, type I error inflation is avoided. However, we note that there is a strong deflation in the type I error rate of the FLGI design. This occurs because the testing procedure used in this section does not include an adjustment similar to the one used with Fisher exact test in Sections 2 and 3. When we look at the mean estimated coefficient for the time trend, we note that CR only slightly underestimates it, having a 40% power to detect it as significantly different from 0. The FLGI design results in a larger underestimation of the time trend coefficient. This underestimation is consistent with that observed in Villar et al^27^ for reasons clarified in Bowden and Trippa.^29^ The power to detect a significant time trend for the FLGI is more than halved compared to CR. Since its estimate is negatively correlated with that of the time trend coefficient, the baseline effect *β*_0_ is also overestimated in both designs, but more severely for the FLGI.

**Table 3:** GLM estimated through MLE with and without Firth correction for T=100, J=5, b=20 in a case of scenario 1 with D=0.24

(I) GLM fitting without correction for CR			
$\hat{\beta_{0}}$	$E\left(\hat{\beta_{i}}\right)$	$E\left(\text{MSE}\right)$	$E\left(p_{value}<0.05\right)$
$\hat{\beta_{0}}$	−0.8684	0.1992	0.5174
$\hat{\beta_{t}}$	0.2610	0.0243	0.4018
$\hat{\beta_{1}}$	0.0070	0.1900	0.0544
(II) GLM fitting with correction for CR			
$\hat{\beta_{0}}$	−0.8370	0.1838	0.5224
$\hat{\beta_{t}}$	0.2509	0.0227	0.4012
$\hat{\beta_{1}}$	0.0067	0.1775	0.0534
(III) GLM fitting without correction for FLGI			
$\hat{\beta_{0}}$	−1.4465	8.9957	0.4110
$\hat{\beta_{t}}$	0.1898	0.0307	0.1844
$\hat{\beta_{1}}$	0.0038	18.2440	0.0142
(IV) GLM fitting with correction for FLGI			
$\hat{\beta_{0}}$	−0.9307	0.3947	0.4670
$\hat{\beta_{t}}$	0.1825	0.0301	0.1858
$\hat{\beta_{1}}$	0.0048	0.7993	0.0456

Abbreviations: CR, complete randomisation; FLGI, forward‐looking Gittins index rule; GLM, generalised linear model; MLE, maximum likelihood estimation; MSE, mean squared error. Results for 5000 trials. True values were assumed to be *β*_0_≈−0.8473, *β*_*t*_≈0.2719 and *β*_*z*_=*β*_1_=0.

Another consequence of the underestimation of the time trend is that complete or quasi‐complete separation is more likely to occur (see Albert and Anderson^30^). This happens for the FLGI, for example, when all the observations of one of the arms are failures (and few in number) and this arm is therefore dropped early from the trial (ie, its allocation probability goes to 0 and never goes above 0 again within the trial).

When this problem occurs in a trial realisation, the maximum likelihood estimates are highly unstable and will not be well defined. This can be observed in the expected mean squared error value for $\hat{\beta_{0}}$ in Table 3 (III) for the FLGI. To address this, we applied Firth's penalised likelihood approach,^31^ which is a method for dealing with issues of separability, small sample sizes, and bias of the parameter estimates (using the R package “logistf”). The use of Firth's approach mitigates the bias due to the separability issue, but it will not correct for the bias caused by the RAR procedure, which is addressed in Bowden and Trippa^29^ or Coad and Ivanova.^32^ To the best of our knowledge, methods that simultaneously adjusts for both sources of bias do not exist. These results suggest that developing bias‐correction methods specially designed for the FLGI within this context could offer improved estimation results than those obtained here. In Table 3 parts (II) and (IV), results of deploying the Firth correction are displayed. These results show an improvement in the estimation of the baseline effect when using the FLGI design: The mean squared error value is significantly reduced, and the average estimate of *β*_0_ is closer to its true value (though it is still overestimated). For the CR design, there is also an improvement. Also, note that the type I error deflation has also been almost fully corrected by the Firth's adjustment in the FLGI design.

To assess the effect on statistical power in Table 4, we replicate the estimation procedure for the case studied in the third row of Table 2 in which we let the treatment effect of arm 1 be positive (ie, *β*_1_≈1.6946) while the overall drift assumed corresponds with *D*=0.16(or *β*_*t*_≈0.1840 and *β*_*z*_=0). The initial success rate in the control arm was equal to 0.3 (ie, *β*_0_≈−0.8473). Because complete (or quasi‐complete) separation affected the FLGI rule in all the scenarios considered here, Table 4 and the following tables only display the results using Firth correction.

**Table 4:** GLM estimated through MLE with Firth correction for T=150, J=5, b=30 in a case of scenario 1 with D=0.16 and β_1_=1.6946

(II) GLM fitting with correction for CR			
$\hat{\beta_{0}}$	$E\left(\hat{\beta_{i}}\right)$	$E\left(\text{MSE}\right)$	$E\left(p_{value}<0.05\right)$
$\hat{\beta_{0}}$	−0.8951	0.1413	0.7194
$\hat{\beta_{t}}$	0.1985	0.0175	0.3262
$\hat{\beta_{1}}$	1.7831	0.1488	0.9994
(IV) GLM fitting with correction for FLGI			
$\hat{\beta_{0}}$	−0.8832	0.3192	0.3364
$\hat{\beta_{t}}$	0.2408	0.0291	0.3062
$\hat{\beta_{1}}$	1.6917	0.4313	0.7394

Abbreviations: CR, complete randomisation; FLGI, forward‐looking Gittins index rule; GLM, generalised linear model; MLE, maximum likelihood estimation; MSE, mean squared error. Results for 5000 trials. True values were assumed to be *β*_0_≈−0.8473, *β*_*t*_≈0.1840, *β*_1_=1.6946 and *β*_*z*_=0.

As expected, the power of a CR design displayed in Table 4 coincides with the value reported in Section 3.3, which using both procedures (ie, adjusting for covariates or hypothesis testing) yields an average value of 99%. The power value of the FLGI design when fitting the generalised linear model is lower than the 80% value reported in Section 3.3 for the case of no time trend (ie, ≈74%). The difference in power levels is explained by the adjustment in Fisher exact test done in that section, which raises power by correcting for the deflation of the type I error rate of the standard Fisher test.

Our results suggest that correctly modelling a time trend and adjusting for separation via Firth correction can safeguard the validity of trial analyses using RAR, that is, by maintaining correct type I error rates and delivering a level of statistical power similar to that obtainable when no trend is present.

### 4.2 Multi‐armed trials

In this section, we consider the case of multi‐armed trials and an instance of scenario 2 or *patient drift*. Also, we shall remove the assumption that the patient covariate or biomarker is unobservable, and allow for the availability of this information before analysing and estimating the corresponding model in (1).

First, we study the case of scenario 2 in which the proportion of biomarker‐positive patients evolves as *q*_*j*_=0.5+(*j*−1)×0.05 for *j*=1,…,10(see Figure 1, middle). We simulated 5000 three‐armed trials of size *T*=200 with *J*=10 and *b*=20. The differential effect of being biomarker positive was assumed to be of 0.3, which corresponds with *β*_*z*_≈1.2528. The initial success rates of all the arms for the biomarker negatives were equal to 0.3 (ie, *β*_0_≈−0.8473).

Table 5 displays the results of the CR, FLGI, and CFLGI designs under the null hypothesis. These results suggest that all designs attain the same power to detect the biomarker effect (as the adaptation is not done using this information, all designs have similar numbers of patients with a positive and a negative biomarker status). More importantly, incorporating patient covariate data into the explicative model dramatically reduces type I error inflation for the FLGI. Although the observed rates are close to 6% and, thus, above the 5% target, they are well below the levels observed without this adjustment (~17%).

**Table 5:** GLM estimated through MLE with Firth correction for T=200, J=10, b=20, K=3 in a case of scenario 2 in which q_j_=[0.5:0.05:0.95]

(II) GLM fitting with correction for CR			
$\hat{\beta_{0}}$	$E\left(\hat{\beta_{i}}\right)$	$E\left(\text{MSE}\right)$	$E\left(p_{value}<0.05\right)$
$\hat{\beta_{0}}$	−0.8527	0.1307	0.6778
$\hat{\beta_{z}}$	1.2597	0.1142	0.9758
$\hat{\beta_{1}}$	−0.0084	0.1305	0.0458
$\hat{\beta_{2}}$	−0.0029	0.1304	0.0486
(IV) GLM fitting with correction for FLGI			
$\hat{\beta_{0}}$	−0.8771	0.1724	0.6740
$\hat{\beta_{z}}$	1.2471	0.1169	0.9702
$\hat{\beta_{1}}$	0.0114	0.2228	0.0598
$\hat{\beta_{2}}$	−0.0097	0.2246	0.0620
(VI) GLM fitting with correction for CFLGI			
$\hat{\beta_{0}}$	−0.8455	0.1338	0.6632
$\hat{\beta_{z}}$	1.2505	0.1200	0.9686
$\hat{\beta_{1}}$	−0.0226	0.1471	0.0558
$\hat{\beta_{2}}$	−0.0196	0.1477	0.0492

Abbreviations: CR, complete randomisation; CFLGI, controlled forward‐looking Gittins index rule; FLGI, forward‐looking Gittins index rule; GLM, generalised linear model; MLE, maximum likelihood estimation; MSE, mean squared error. Results for 5000 trials. True values were assumed to be *β*_0_≈−0.8473, *β*_*z*_≈1.2528 and *β*_*t*_=*β*_1_=*β*_2_=0.

In Table 6, we examine the effect on power by replicating the previously described scenario but allowing for the experimental arm 1 to have an effect for all patients of 0.2 (ie, *β*_1_=0.8473). These results show how the power to detect the treatment effect in arm 1 with an FLGI design is almost halved compared to that attained by a traditional CR design. Yet the CFLGI improves on the power level of the CR design by approximately 15%. Also note that the type I error rate for arm 2 appears to be deflated for the FLGI and CFLGI designs.

**Table 6:** GLM estimated through MLE with Firth correction for T=200, J=10, b=20, K=3 in a case of scenario 2 in which q_j_=[0.5:0.05:0.95]

(II) GLM fitting with correction for CR			
$\hat{\beta_{0}}$	$E\left(\hat{\beta_{i}}\right)$	$E\left(\text{MSE}\right)$	$E\left(p_{value}<0.05\right)$
$\hat{\beta_{0}}$	−0.8816	0.1324	0.7078
$\hat{\beta_{z}}$	1.3006	0.1239	0.9726
$\hat{\beta_{1}}$	0.9355	0.1549	0.6954
$\hat{\beta_{2}}$	−0.0032	0.1356	0.0516
(IV) GLM fitting with correction for FLGI			
$\hat{\beta_{0}}$	−1.1635	0.5391	0.3994
$\hat{\beta_{z}}$	1.3492	0.1300	0.9762
$\hat{\beta_{1}}$	1.1300	0.5845	0.3672
$\hat{\beta_{2}}$	0.0041	0.7740	0.0246
(VI) GLM fitting with correction for CFLGI			
$\hat{\beta_{0}}$	−0.8861	0.1378	0.6966
$\hat{\beta_{z}}$	1.3127	0.1243	0.9718
$\hat{\beta_{1}}$	0.8862	0.2077	0.7718
$\hat{\beta_{2}}$	−0.2487	0.5027	0.0288

Abbreviations: CR, complete randomisation; CFLGI, controlled forward‐looking Gittins index rule; FLGI, forward‐looking Gittins index rule; GLM, generalised linear model; MLE, maximum likelihood estimation; MSE, mean squared error. Results for 5000 trials. True values were assumed to be *β*_0_≈−0.8473, *β*_*z*_≈1.2528 and *β*_1_=0.8473, *β*_*t*_=*β*_2_=0.

These results suggest that fitting a model that includes a time trend after having used a RAR rule can protect against the type I error inflation caused by *patient drift* as long as the patient covariate information is observable and available to adjust for. However, the power level attained by covariate adjustment is considerably less than that attained by a design that protects the allocation to the control arm.

Furthermore, these results fail to illustrate the learning‐earning trade‐off that characterises the choice between a CR and a RAR procedure and the reasons why the FLGI could be desirable to use from a patient benefit perspective (despite the power loss and the type I error inflation potential). The traditional CR design, which maximises learning about all arms, yields an average number of successfully treated patients (or ENS) of 116.83 when *p* ^∗^ remains fixed by design at 1/3. The FLGI design, on the other hand, is almost optimal from a patient benefit perspective, achieving an ENS value of 135.21, 15.73% higher than with CR, and it achieves this by skewing *p* ^∗^ to 0.7783. Finally, the CFLGI is a compromise between the 2 opposing goals that improves on the power levels attained by a CR design and also on its corresponding ENS value (though is below the value that could be attained with the unconstrained FLGI rule) by attaining an ENS value of 126.32, 8.12% more than with a CR design, and a *p* ^∗^ of 0.5618.

## 5 DISCUSSION

Over the past 65 years, RCTs have become the gold standard approach for evaluating treatments in human populations. Their inherent ability to protect against sources of bias is undoubtedly one of their most attractive features and is also the reason that many are unwilling to recommend the use of RAR rules, feeling that this would be a “step in the wrong direction.”^33^ Recently, Thall et al^3^ have suggested that a severe type I error inflation could occur if RAR is used under the presence of an unaccounted for time trend. The temporal heterogeneity of the study population is a reasonable concern for trials in rare diseases as they tend to last several years. However, there remains a strong interest in the medical community to use RAR procedures in this very setting.^34^ The considerable patient benefit advantages offered by non‐myopic RAR procedures can increase trial acceptability amongst both patients and physicians and enhance patient enrollment. Additionally, in the multi‐armed case, the modified non‐myopic procedures offer also increased statistical efficiency when compared with a traditional RCT.

We have assessed the level of type I error inflation of several RAR procedures by creating scenarios that are likely to be a concern in modern clinical trials that have a long duration. Our results suggest that the magnitude of the temporal trend necessary to seriously inflate the type I error of the *patient benefit‐oriented* RAR rules needs to be of an important magnitude (ie, change larger than a 25% in its outcome probability) to be a source of concern. This supports the conclusion of Karrison et al^5^ in a group sequential design context. However, we also conclude that certain RAR rules are effectively immune to time trends, specifically those that are power oriented such as the CFLGI rule^19^ or the RSIHR rule.^21^ This suggests that when criticising the use of RAR in real trials, one must be careful not to include all RAR rules in the same class, as they have markedly different performances in the same situation.

In addition, we have recommended 2 different procedures that can be used in an RAR design to protect for type I error inflation. For 2‐armed trials, the use of a randomisation test (instead of traditional tests) preserves type I error under any type of unknown temporal trend. The use of randomisation as a basis for inference provides robust assumption‐free testing procedures, which depend explicitly on the randomisation procedure used, be it a RAR procedure or not. The cost of this robustness may be a computational burden and a reduction in statistical power (although most patients are still allocated to a superior arm when it exists if using the FLGI procedures). This particular feature highlights the need to develop computationally feasible testing procedures that are specifically tailored to the behaviour of a given RAR rule. For example, as pointed out by Villar et al,^27^ bandit‐based rules such as the FLGI are extremely successful at identifying the truly best treatment but, as a direct result, often cannot subsequently declare its effect “significant” using standard testing methods.

For multi‐armed clinical trials, protecting allocation to the control group (the recommended procedure) preserves the type I error while yielding a power increase with respect to a traditional CR design. However, despite rules such as the CFLGI being more robust to a time trend effect, they also offer a reduced patient benefit in the case that there is a superior treatment, when compared to the *patient benefit‐oriented* RAR rules such as the FLGI.

Finally, we also assessed adjustment for a time trend both as an alternative protection procedure against type I error inflation and to highlight estimation problems that can be encountered when an RAR rule is implemented. Our conclusion is that adjustment can alleviate the type I error inflation of RAR rules (if the trend is correctly specified and the associated covariates are measured and available). However, for the multi‐armed case, this strategy attains a lower power than simply protecting the allocation to the control arm. Furthermore, the technical problem of separation also complicates estimation after the *patient benefit‐oriented* RAR rules have been implemented and severely impact the power to detect a trend compared to an CR design.

Large unobserved time trends can result in an important type I error inflation, even if using restricted randomisation algorithms that seek to balance the number of patients in each treatment group.^28^ ^, Section 6.10;^ 35 Therefore, an important recommendation of this work is that in the design stage of a clinical trial, when a RAR procedure is being considered, a similar detailed evaluation of type I error inflation and bias as done in this paper is performed to choose a suitable RAR procedure for the trial at hand.

Further research is also needed to assess the potential size of time trends through careful reanalysis of previous trial data (as Karrison et al^5^ do with data from Kalish and Begg^36^). Our results suggest that even in the case of a large time trend being a realistic concern, there are still some RAR rules that, both for the 2‐armed and the multi‐armed case, remain largely unaffected in all the cases we have considered. Of course, these rules offer increased patient benefit properties when compared to a traditional CR design but reduced when compared to the *patient benefit‐oriented* RAR rules.

Another area of future work is to explore the combination of randomisation tests with stopping rules in a group sequential context, specifically for the FLGI. Additionally, techniques for the efficient computation of approximate randomisation tests for the FLGI could be studied, similar to those explored in Plamadeala and Rosenberger.^37^
